# Bistable auto-aggregation phenotype in *Lactiplantibacillus plantarum* emerges after cultivation in in vitro colonic microbiota

**DOI:** 10.1186/s12866-021-02331-x

**Published:** 2021-10-05

**Authors:** Julia Isenring, Annelies Geirnaert, Christophe Lacroix, Marc J. A. Stevens

**Affiliations:** 1 Laboratory of Food Biotechnology, Institute of Food, Nutrition and Health, ETH Zurich, Schmelzbergstrasse 7, 8092 Zürich, Switzerland; 2 Institute for Food Hygiene and Safety, University of Zürich, Zurich, Switzerland

**Keywords:** Auto-aggregation, *Lactiplantibacillus plantarum*, In vitro gut microbiota, Adaptation, Transcriptome, DNA inversion

## Abstract

**Background:**

Auto-aggregation is a desired property for probiotic strains because it is suggested to promote colonization of the human intestine, to prevent pathogen infections and to modulate the colonic mucosa. We recently reported the generation of adapted mutants of *Lactiplantibacillus plantarum* NZ3400, a derivative of the model strain WCFS1, for colonization under adult colonic conditions of PolyFermS continuous intestinal fermentation models. Here we describe and characterize the emerge of an auto-aggregating phenotype in *L. plantarum* NZ3400 derivatives recovered from the modelled gut microbiota.

**Results:**

*L. plantarum* isolates were recovered from reactor effluent of four different adult microbiota and from spontaneously formed reactor biofilms. Auto-aggregation was observed in *L. plantarum* recovered from all microbiota and at higher percentage when recovered from biofilm than from effluent. Further, auto-aggregation percentage increased over time of cultivation in the microbiota. Starvation of the gut microbiota by interrupting the inflow of nutritive medium enhanced auto-aggregation, suggesting a link to nutrient availability. Auto-aggregation was lost under standard cultivation conditions for lactobacilli in MRS medium. However, it was reestablished during growth on sucrose and maltose and in a medium that simulates the abiotic gut environment. Remarkably, none of these conditions resulted in an auto-aggregation phenotype in the wild type strain NZ3400 nor other non-aggregating *L. plantarum*, indicating that auto-aggregation depends on the strain history. Whole genome sequencing analysis did not reveal any mutation responsible for the auto-aggregation phenotype. Transcriptome analysis showed highly significant upregulation of LP_RS05225 (*msa*) at 4.1–4.4 log_2_-fold-change and LP_RS05230 (*marR*) at 4.5–5.4 log_2_-fold-change in all auto-aggregating strains compared to non-aggregating. These co-expressed genes encode a mannose-specific adhesin protein and transcriptional regulator, respectively. Mapping of the RNA-sequence reads to the promoter region of the *msa*-*marR* operon reveled a DNA inversion in this region that is predominant in auto-aggregating but not in non-aggregating strains. This strongly suggests a role of this inversion in the auto-aggregation phenotype.

**Conclusions:**

*L. plantarum* NZ3400 adapts to the in vitro colonic environment by developing an auto-aggregation phenotype. Similar aggregation phenotypes may promote gut colonization and efficacy of other probiotics and should be further investigated by using validated continuous models of gut fermentation such as PolyFermS.

**Supplementary Information:**

The online version contains supplementary material available at 10.1186/s12866-021-02331-x.

## Background

Consumption of probiotics increased steadily over the past years and their application was recommended against a range of gastrointestinal tract related diseases [1]. Probiotics are defined as “live microorganisms that, when administered in adequate amounts, confer a health benefit on the host “[2]. To exert a health benefit, a probiotic should exhibit certain properties such as survival during gastrointestinal passage, delivery of high viable-cell numbers to the colon and a certain degree of colonization in the gut environment [3, 4]. Successful colonization in the intestine is mediated via adherence to the intestinal epithelium or solid particles.

Auto-aggregation occurs between genetically identical cells and co-aggregation between genetically different cells [5]. Auto-aggregation is a well-studied phenomenon that leads to the formation of a community structure that facilitates interaction and communication between cells, genetic exchange, adherence and colonization in different environments [6–11]. Auto-aggregation ability correlates positively with adherence to human epithelial cell lines and the ability to co-aggregate with *Listeria monocytogenes*, *Staphylococcus aureus* and pathogenic *Escherichia coli* [8, 12–16]. Auto-aggregation is frequently assessed as feature in studies evaluating putative probiotic strains [17–19].

Although auto-aggregation is a common phenomenon, there is still no complete understanding of underlying mechanisms and triggers, possibly impeded by species-specific differences. Triggering factors of auto-aggregation so far identified include intestinal, nutritive, chemical, and oxidative stresses, changes in temperature and nutrient availability [20–25]. Several auto-aggregation mechanisms have been reported, including cell-surface properties, −structures and -enzymes. Cell surface hydrophobicity for example is positively linked to auto-aggregation [14, 26, 27]. Molecules involved in auto-aggregation, so called autoagglutinins, include cell-surface proteins, exopolysaccharides, carbohydrates, glycoproteins, teichoic and lipoteichoic acid secreted proteins that act as aggregation promoting factors [7, 23, 28, 29].

Auto-aggregation was reported for *Lactobacillus* spp. strains isolated from distinct environments like the piglet, chicken and murine gastrointestinal tract, vaginal tract, dairy and fermented foods [12, 30–37]. However, not much is known about bacterial auto-aggregation and its role in the human intestinal tract. *Lactiplantibacillus plantarum* WCFS1 is a model strain for probiotic lactobacilli. It harbors possible genetic predisposition for auto-aggregation in form of a serine/threonine rich domain in LP_RS01260 with affinity to mucin which was identified to be involved in auto-aggregation of *L. plantarum* NCIMB 8826, the mother strain of WCFS1 [38, 39]. However, there is no description of a WCFS1 auto-aggregation phenotype yet. Recently, we supplemented *L. plantarum* NZ3400, a derivative strain of WCFS1, to different in vitro human colonic microbiota, continuously cultivated in the PolyFermS model in the context of an evolutionary engineering experiment [40]. *L. plantarum* NZ3400 derivatives were recovered from the microbiota and phenotypically and genotypically characterized. Here, we describe the emerge of an auto-aggregation phenotype in a non-aggregating strain after exposure to human colon conditions. The novel phenotype was characterized, and transcriptome analysis was performed to investigate the underlying mechanisms.

## Results

### Development of a high-throughput screening for auto-aggregation

**Fig. 1:**
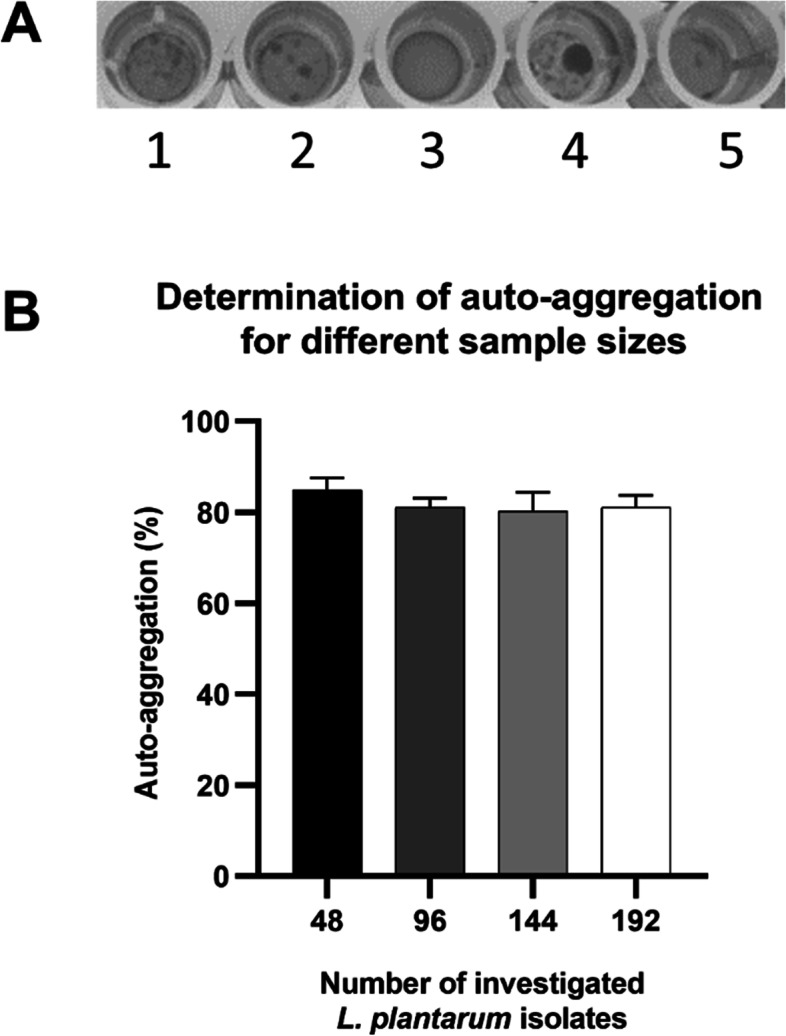
Visual auto-aggregation detection and determination of the number of colonies to investigate auto-aggregation. **A** 96-well plate well bottom appearance of non-aggregating (well 3) and auto-aggregating (well 1–2,4–5) *L. plantarum* isolates. **B** Percentage of auto-aggregation after testing different numbers of *L. plantarum* colonies

A high-throughput screening method based on a yeast-agglutination assay was set-up to detect auto-aggregating *L. plantarum* strains [41]. Microscopic investigation of 96 *L. plantarum* cultures grown in a 96-well tissue culture test plate revealed that auto-aggregating *L. plantarum* cultures produced a sediment of white clumps, while non-aggregating cultures formed a homogenous white layer at the bottom of the wells (Fig. 1A). Therefore, detection of clumps can be used to distinguish rapidly between auto-aggregating and non-aggregating strains.

To determine how many *L. plantarum* isolates have to be analyzed to obtain a reliable percentage of the auto-aggregation subpopulation, different sets of colonies isolated from a single reactor and time point were visually assigned as aggregating or not. Testing 48, 96, 144 and 192 colonies did not significantly change aggregation percentage (Fig. 1B). Therefore, 48 colonies were investigated in all further experiments to determine auto-aggregation percentages.

### *L. plantarum* auto-aggregation increases in biofilm, during starvation and during cultivation in in vitro colonic microbiota

**Fig. 2:**
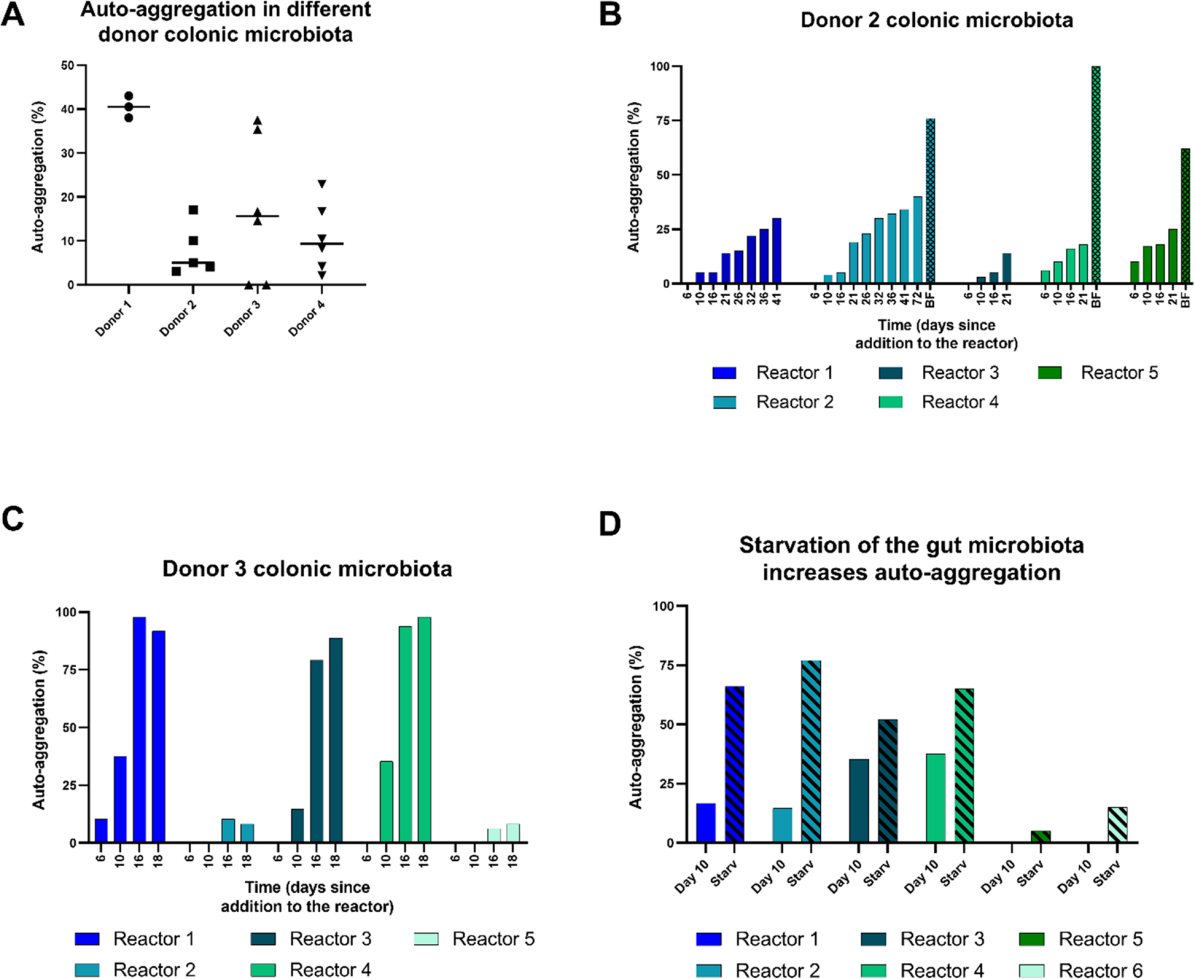
Influence of different microbiota, biofilm and starvation on *L. plantarum* auto-aggregation percentage. *L. plantarum* strains were recovered from four different in vitro human adult colonic microbiota. **A** Auto-aggregation percentage of *L. plantarum* strains recovered after 10 days of cultivation in reactors inoculated with colonic microbiota from four different donors. Each data point represents auto-aggregation percentage in one reactor. **B** Auto-aggregation percentage of *L. plantarum* strains recovered from five reactors inoculated with microbiota of donor 2 over time and from reactor biofilms (BF). **C** Auto-aggregation percentage of *L. plantarum* recovered from five reactors containing colonic microbiota of donor 3. **D** Auto-aggregation percentage at the day before and one day after starvation of the microbiota of donor 4 (Starv)

To investigate if auto-aggregation is microbiota-dependent, *L. plantarum* NZ3400 was supplemented to four PolyFermS models inoculated with different colonic microbiota and recovered after 10 days. Auto-aggregation was detected in *L. plantarum* isolates recovered from all four microbiota at levels between 8 and 41% (Fig. 2A). Auto-aggregation percentage steadily increased over time in five different reactors containing microbiota of donor 2, reaching approximately 15 and 30% after 21 and 42 days post supplementation, respectively (Fig. 2B). An increase in auto-aggregation over time was also observed for donor 3 microbiota. It was more pronounced compared to donor 2 microbiota in the reactors 1, 3 and 4 with 88–89% aggregation 18 days post *L. plantarum* supplementation, but lower in the reactors 2 and 5 with only 8% aggregation after 18 days (Fig. 2C).

Further, biofilms spontaneously formed on the reactor walls were collected at the end of fermentations. The auto-aggregation percentage of *L. plantarum* strains from biofilms was at least two times higher than from reactor effluent at the final day of fermentation (Fig. 2B), suggesting a role of auto-aggregation in biofilm establishment. However, no correlation was found between auto-aggregation percentage and *L. plantarum* colonization level in the reactor (data not shown). Since aggregation might facilitate the formation of biofilms, biofilm formation ability of the wild type and the aggregating, isogenic strain PA4_02 on polystyrene was assessed using crystal violet and optical density measurement. Strains were grown compared in MRS and minimal medium containing different carbon sources. The aggregating strain PA4_02 showed clearly increased biofilm formation compared to the wild type when grown in minimal medium supplemented with maltose but not in any other conditions (Additional file 1: Figure S1).

Auto-aggregation is suggested to be linked to cell-proximity during nutrient exchange or to nutritive stress [23]. Therefore, starvation of colonic microbiota was simulated by interrupting the medium inflow for 24 h in six TRs containing microbiota of donor 4. Starvation led to accumulation of isobutyrate, isovalerate and valerate, which indicates enhanced protein fermentation (Additional file 2: Figure S2). Auto-aggregation percentage increased during starvation in all reactors (Fig. 2D). Moreover, after starvation, auto-aggregation was even detected in reactors where no auto-aggregation was observed before the starvation period (Fig. 2D, reactor 5 and 6). Simultaneously, *L. plantarum* viable cell counts decreased by 1–2 logs during starvation (data not shown).

### Bistability of auto-aggregation phenotype depends on nutrient availability

**Fig. 3:**
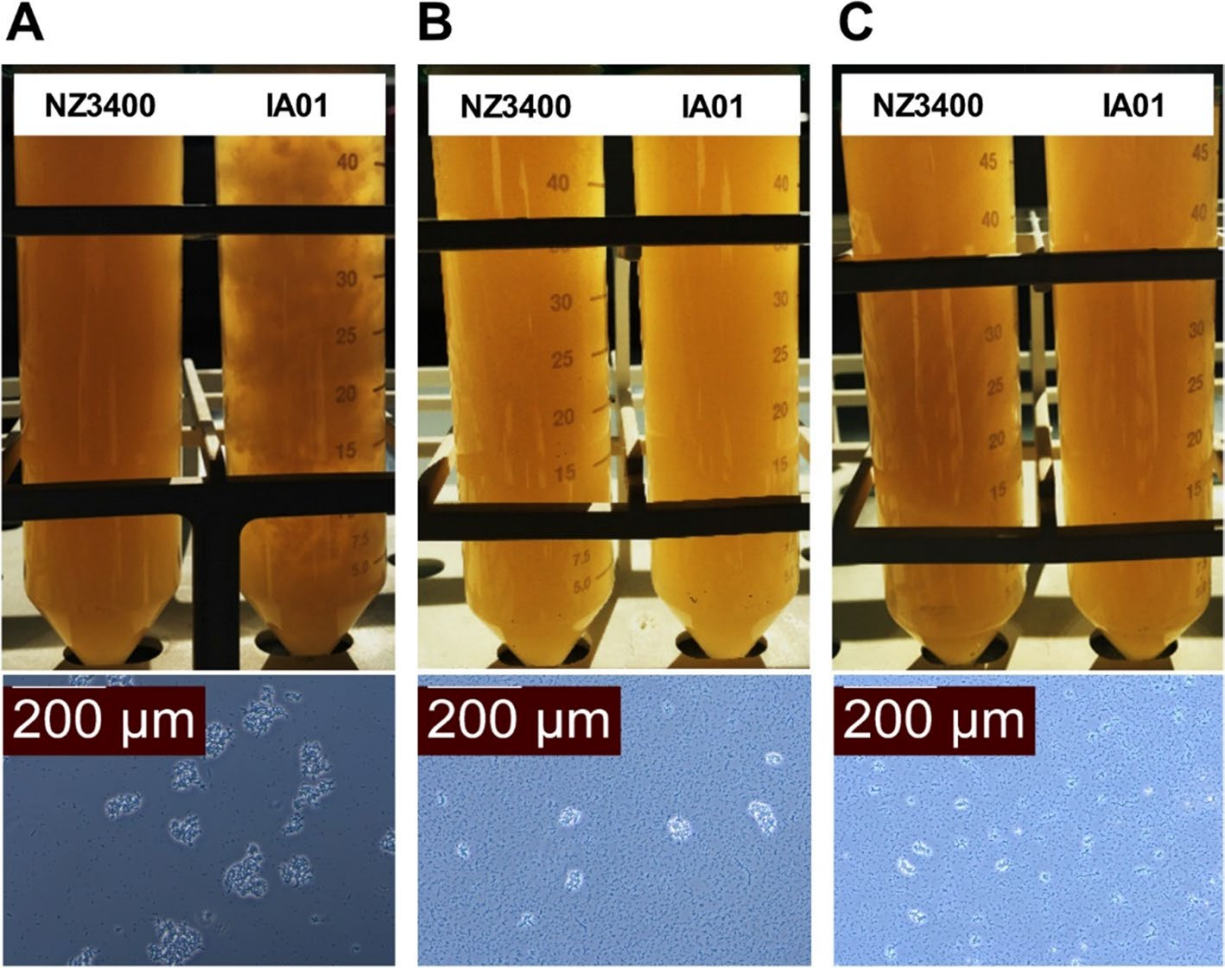
Auto-aggregation phenotype diminishes during consecutive cultivation in MRS broth. Visual comparison between *L. plantarum* NZ3400 (left tube) and IA01 (right tube) and microscopic images of IA01 after the first (**A**), second (**B**) and third (**C**) consecutive culture in MRS broth. Results are representative for 15 tested *L. plantarum* cultures with auto-aggregation phenotype

To investigate the stability of the auto-aggregation phenotype, fifteen *L. plantarum* NZ3400 derivatives with auto-aggregation phenotype, recovered from all four microbiota, were serially cultured in MRS medium daily for 3 days. All strains still exhibited a strong auto-aggregation phenotype that was clearly visible after the first overnight culture (Fig. 3A). Auto-aggregation was not visually detectable anymore after the second overnight culture, yet small aggregates were still observed microscopically (Fig. 3B). Finally, aggregates were only barely detectable microscopically in the third culture (Fig. 3C). Conclusively, auto-aggregation is instable under nutrient-rich cultivation conditions, hinting again towards a role of nutrient availability in auto-aggregation.

Thereafter, *L. plantarum* strains that lost their aggregation capacity upon consecutive cultivation in MRS medium were grown in a minimal medium supplemented with different carbon sources and in EMS, a medium that simulates the abiotic environment of the reactor gut microbiota [40]. *L. plantarum* strains IA01, PA4_02 and PA1.2_01 lost their auto-aggregation capacity in repeated MRS cultures but recovered this capacity when regrown in EMS medium and in minimal medium containing sucrose or maltose, but not in the same medium with glucose as sole carbon source. Remarkably, *L. plantarum* NZ3400 and four isolates (Table 1) recovered from the modelled microbiota of donor 1 and 2 that never showed auto-aggregation, did not auto-aggregate in any of the tested conditions. These results show that different nutrient conditions as such do not result in an auto-aggregation phenotype in *L. plantarum* NZ3400 and its derivatives, but that auto-aggregation is only observed in strains that have been exposed to the gut microbiota.

**Table 1:** *L. plantarum* NZ3400 derivative strains recovered from human in vitro microbiota

Strain	Description	Auto-aggregation	Reference
IA01	NZ3400 derivative, Cm^R^, C979T in LP_RS14990, isolated from in vitro fermentation	+	Isenring et al., 2021 [40]
IA01_t0_	Strain IA01 grown after one overnight culture in MRS	+	This study
IA01_t4_	Strain IA01 grown after four consecutive cultures in MRS	–	This study
PA4_02	NZ3400 derivative, Cm^R^, isolated from in vitro fermentation	+	Isenring et al., 2021 [40]
PA1.2_01	NZ3400 derivative, Cm^R^, C979T in LP_RS14990 and G382A in LP_RS01530, isolated from in vitro fermentation	–	Isenring et al., 2021 [40]
IA10	NZ3400 derivative, Cm^R^, C569A in LP_RS14255, isolated from in vitro fermentation	–	Isenring et al., 2021 [40]
PA2_04	NZ3400 derivative, Cm^R^, C837A in LP_RS15205, isolated from in vitro fermentation	–	Isenring et al., 2021 [40]
PA2_06	NZ3400 derivative, Cm^R^, C837A in LP_RS15205, isolated from in vitro fermentation	–	Isenring et al., 2021 [40]

### Genome and transcriptome analyses of auto-aggregating *L. plantarum* isolates

The possibility to reestablish auto-aggregation only in isolates that were exposed to modelled colonic microbiota suggests a lasting genomic modification responsible for the phenotype. However, whole genome sequencing analysis did not reveal any shared single nucleotide polymorphisms nor genetic reorganization in seven auto-aggregating isolates compared to NZ3400. Moreover, three thereof did not harbor any mutation and were thus isogenic to *L. plantarum* NZ3400 (data not shown). Because genome sequences did not provide an explanation for the auto-aggregation phenotype, transcriptome analysis using RNA sequencing was performed to identify genes possibly involved in the auto-aggregation phenotype.

The transcriptome of the wild type strain NZ3400 was compared to that of the strain IA01_t0_ during growth in MRS, conditions under which AI01 aggregates and NZ3400 does not. The comparison revealed 192 significantly up- and 289 downregulated genes in IA01 compared to NZ3400 (FDR < 0.05; Additional file 3: Table S1). In addition, IA01 was grown in four consecutive overnight cultures in MRS, which led to loss of the auto-aggregation phenotype, resulting in the strain IA01_t4_. Comparison of aggregating IA01_t0_ and non-aggregating IA01_t4_ resulted in 583 significantly up- and 632 downregulated genes in IA01_t0_ (Additional file 4: Table S2). Further, 94 up- and 123 downregulated genes were shared in both comparisons, being primary candidate genes for involvement in auto-aggregation. To narrow down these candidate genes, RNA sequencing data of the auto-aggregating strain PA4_02 and the wild type strain NZ3400 were compared. A total of 138 genes were significantly upregulated and 143 were significantly downregulated in PA4_02 (Additional file 5: Table S3). Combining all three comparisons revealed 50 significantly up- and 62 downregulated genes in auto-aggregating strains versus non-aggregating strains (Additional file 6: Table S4). Thereof, only genes with a │log_2_ ratio│ > 1 amongst all three comparisons were considered as possible candidate genes involved in auto-aggregation (Table 2). Up-regulated genes in this data set encoded for two hypothetical proteins, three proteins with domains of unknown function (DUF), a haloacid dehalogenase-like hydrolase (HAD), the Multiple Antibiotic Resistance Regulator (MarR), and a mucin-binding protein (MucBP) domain (Table 2). The latter is known to be a mannose-specific adhesion protein encoded by *msa* [42]. The MarR family transcriptional regulator (LP_RS05230, *marR*) and the MucB domain-containing protein (LP_RS05225, *msa*) were strongest regulated by far with a log-fold change of 4.5–5.4 and 4.1–4.4, respectively (Table 2). The *marR* gene is located 11 bp downstream of the *msa* gene and in the same direction, suggesting that they belong to one operon.

**Table 2:** Genes significantly up- and down-regulated (│Ratio(log_2_)│ > 1) in auto-aggregating compared to non-aggregation strains*

ORF	Product	Ratio (log_**2**_)		
		PA4_02^**a)**^	IA01_**t0**_^**b)**^	IA01_**t0**_^**c)**^
**Upregulated**				
LP_RS05230	MarR family transcriptional regulator	−5.17	−4.54	− 5.37
LP_RS05225	MucBP domain-containing protein	−4.23	−4.10	− 4.42
LP_RS02885	Hypothetical protein	−2.17	−1.54	−1.25
LP_RS10335	DUF4355 domain-containing protein	−2.04	−1.53	−1.29
LP_RS02870	DUF4355 domain-containing protein	−1.90	−1.18	− 1.22
LP_RS10300	Hypothetical protein	−1.55	−1.03	− 1.07
LP_RS14830	HAD family hydrolase	−1.52	−1.11	− 1.28
LP_RS10310	DUF3168 domain-containing protein	−1.49	−1.58	− 1.20
**Downregulated**				
LP_RS01470	Hypothetical protein	1.05	1.27	1.22
LP_RS00795	Cna B-type domain-containing protein	1.08	1.02	1.04

^a^Auto-aggregating PA4_02 compared to the wild type NZ3400

^b^Auto-aggregating IA01t_0_ compared to IA01t_4_

^c^Auto-aggregating IA01t_0_ compared to the wild type NZ3400

*phage-related genes were omitted. ORF: open reading frame

Further, 2 genes encoding a hypothetical protein and Can B-type domain-containing protein, were significantly down-regulated in all three analyses (Table 2). However, the magnitude of change (maximum of 1.27 log-fold change) was small compared to the *msa-marR* operon (Table 2).

### DNA inversion predominates in auto-aggregating *L. plantarum* strains

The very strong regulation of the *msa-marR* operon makes this operon the primary candidate responsible for the auto-aggregation phenotype. The *msa* promoter region contains two repeats that can invert, thereby changing the promoter activity of the operon [41]. To test whether such inversion occurred in auto-aggregating strains, the RNAseq reads were plotted on the genome of WCFS1 and checked carefully for any genetic reorganization in the *msa* region. Indeed, an alternative junction was identified in all samples of the aggregating strains IA01_t0_ and PA4_02, exactly at the repeat in the promoter of *msa*. This new junction was not identified in the transcriptome samples from the wild type, IA01_t4_, nor in the samples of a previously constructed non-aggregating *lamC* knockout strain *L. plantarum* ΔLP_RS14990 [40] (Isenring, unpublished results).

Next, we quantified the occurrence of the inverse promoter using the RNAseq data reads. The ratio between the native and the inverted promoter was 8.3 ± 1.2 in the wild type and 9.5 ± 1.4 in the ΔLP_RS14990 strain, showing high prevalence of the native promoter in both non-aggregating strains. In contrast, the auto-aggregating strains A01_t0_ and PA4_02 had a significantly lower ratio of 0.56 ± 0.04 and 0.54 ± 0.07 than the wild type strain and ΔLP_RS14990 (*p* = 0.001). Strain IA01_t4_ lost its auto-aggregating phenotype and had a significantly lower a ratio of 5.3 ± 0.9 than the wild type (*p* = 0.03) suggesting that the inverted promoter is still present in strains that have lost the phenotype recently.

Last, we investigated whether the inverted promoter region is already present in a subpopulation of the wild type strain and in the non-aggregating strain IA10 (Table 1). A PCR specific for the native promoter resulted in amplification for the wild type strain NZ3400, strain IA10 and for both aggregating strains PA4_02 and AI01 (Additional file 7: Figure S3), supporting that the native promoter is present in all *L. plantarum* cultures. However, the PCR specific for the inverted promoter did not result in amplification for the wild type and IA10 strains but produced a clear amplicon in the cultures of PA4_02 and IA01.

## Discussion

Auto-aggregation is a desired property for probiotics which is associated with enhanced colonization, inhibition of pathogenic infections and immunomodulation of the intestinal mucosa [12, 13, 28, 43, 44]. In this study, we describe for the first time the emerge of an auto-aggregating phenotype in the non-aggregating *L. plantarum* NZ3400 after exposure to modelled human colonic microbiota. Percentage of auto-aggregation was highest for *L. plantarum* isolated from reactor biofilms. Biofilm formation can be facilitated by auto-aggregation [5, 45], however, quantification of biofilm formation on a polystyrene surface via crystal violet showed that biofilm formation of the aggregating strain PA4–02 was only enhanced compared to the wild type in minimal medium containing maltose. Aggregates were reported to form a multi-species cell-network that facilitates nutrient exchange, provides protection and creates a novel niche that aids microbiota colonization [23, 46]. *L. plantarum* cells in aggregates might therefore be able to survive better during starvation. It should be noted that starvation did not induce changes in propionate and butyrate concentrations and glucose, galactose and lactate were not detectable in both, control, and starved conditions. Therefore, these factors and the pH, which was kept constant during fermentation, are not explaining the effects observed on aggregation.

Transcriptome analysis of the wild type strain and auto-aggregating *L. plantarum* strains showed that the genes encoded by LP_RS05225 (*msa*) and LP_RS05230 (*marR*) were both highly overexpressed in the auto-aggregation phenotype. LP_RS05230 (previously known as lp_1230) is a transcriptional regulator that is located 11 base pairs downstream of LP_RS05225. In silico analysis of the genes showed that they form a bicistronic operon [41, 42], which is also supported by our data. The *msa* gene was identified as mannose-specific adhesin gene and is responsible for the agglutination of *L. plantarum* with *Saccharamyces cerevisiae* [41, 42]. Mannose-specific adherence mediates adherence of *L. plantarum* to human colonic cell line HT-29, epithelial Caco-2 cells and intestinal mucus in rats and thus assists in intestinal colonization [41, 47, 48]. In addition, pathogens such as *Vibrio cholerae*, *Salmonella* or *Pseudomonas aeruginosa* bind to mannose-containing glycoconjugates on the host intestinal surface [49–51]. Adherence of *L. plantarum* by mannose-specific adhesins to such glycoconjugates might therefore prevent binding of the pathogens. Furthermore, mannose-specific adhesins reduce pathogen colonization due to co-aggregation with mannose-containing surface structures of the pathogen [52–54]. Hence, activation of *msa* may be a desirable property for probiotics.

Identification of *marR* and *msa* did not reveal the mechanism sustaining the auto-aggregation phenotype, since genome sequencing did not reveal any mutation or other variation in the auto-aggregating strains. However, plotting RNAseq reads on the wild type genome revealed two novel DNA-junctions in the promoter region of LP_RS05225, that apparently could only be detected with the high read numbers produced in RNAseq experiments and the dedicated breseq software with a low detection limit for identification of variations in the genome [55]. The junction is identical to a previously reported inversion that is responsible for a strong yeast-agglutination phenotype in *L. plantarum* WCFS1, the parental strain of NZ3400 [41]. The inversion causes prevention of a stem-loop structure increasing both, transcription and translation of the *msa* gene. The inversion was predominant in aggregation cultures and still more present in IA01_t4_ than in non-aggregating stains. The occurrence of the aggregating phenotype over several generations can be explained by the presence of this relatively stable inversion. Moreover, the detected remains of the inversion in cultures that lost their aggregation phenotype may explain the rapid reverse of these cultures to an aggregating phenotype under selected conditions.

Aggregation was activated in absence of detectable glucose in fermentation reactors and in minimal medium without glucose. In addition, aggregation disappeared in glucose containing MRS medium. This suggests a role of glucose mediated catabolite repression via the canonical regulatory carbon catabolite protein A (CcpA) [56]. CcpA binds to catabolite response elements (*cre*) in the proximity to the regulated promoter, thereby regulating gene expression [57]. However, involvement of CcpA seems unlikely because reestablishment of auto-aggregation also occurred in the glucose-containing EMS medium. Further, no cre-elements were identified in the promoter region of *msa* and transcriptome analysis of a *L. plantarum* WCFS1 ccpa-knockout strain did not reveal differential expression in LP_RS05225 and LP_RS05230 [58]. Other regulators might be involved in the msa promoter inversion, yet the exact regulation remains unclear.

The observed loss and reestablishment of the auto-aggregation phenotype could be explained by phase variation, which leads to an heterogenous culture via an ON/OFF switch [59]. DNA inversions cause phase-variable surface protein expression [60–66] partially via genetic modifications by the activity of recombinases [60, 61, 67–70]. Indeed, recombinases lead to DNA inversion in *Bacteroides fragilis* resulting in phase-variable expression of surface proteins responsible for auto-aggregation [71]. The higher expression of the recombinase RecT (Additional file 6: Table S4) in auto-aggregating strains might induce DNA inversion in the upstream region of *msa*. However, classical phase variation in the sense of an ON-OFF switch does not explain the slow disappearance of auto-aggregation during three consecutive MRS cultures.

Alternatively, growth in MRS medium might select for the native promoter, which is shown by the presence of the native orientation in the aggregating strains IA01 and PA4_02 (Additional file 7: Figure S3). Overexpression of the mannose-specific adhesin is toxic in *L. plantarum* 299v, but not WCFS1, yet might lead to slower growth [41], explaining the vanishing of the auto-aggregating phenotype. Growth in MRS medium can then select for the fast-growing, non-aggregating fraction resulting in outcompeting the aggregating fraction during consecutive culturing. Slower growth of *L. plantarum* on sucrose, maltose [58] and EMS (data not shown) would allow outgrowth of the auto-aggregating fraction. Strong supporting evidence for this explanation is the difference in inverse and native promoter prevalence between the wild type and auto-aggregating variants.

The presence of both promoter orientations in aggregating strain cultures shows that an aggregating subculture proliferates in the fermenter. The inverse promoter orientation could not be detected in non-aggregating strains, suggesting strongly that the inversion is not present in non-aggregating cultures but is rather a relatively rare event and must be selected for to become visible. However, whether the inversion occurs spontaneously or is induced by the fermenter environment remains unclear.

## Conclusion

We showed that cultivation of *L. plantarum* in a modelled in vitro human colonic microbiota leads to an auto-aggregating subpopulation. This novel auto-aggregating phenotype is caused by a high expression of the mannose-specific adhesion gene *msa*. Both, auto-aggregation and mannose-mediated adhesion are desired properties of probiotics. Our data demonstrate that the PolyFermS fermentation model is suitable to detect and select for novel phenotypes related to the intestinal environment. This could further be applied to elucidate phenotypic adaptations and their underlying mechanisms of both probiotics and gut pathogens.

## Methods

### Strains and growth conditions

*L. plantarum* NZ3400, a WCFS1 derivative harboring a chloramphenicol resistance cassette in a neutral locus on the chromosome, was used as wild type strain [72]. NZ3400 derivative strains were recovered from in vitro human colonic microbiota after cultivation for up to 100 generations as described previously [40] (Table 1).

*L. plantarum* strains were cultivated in De Man, Rogosa and Sharpe (MRS, Labo-Life Sàrl, Pully, Switzerland) broth at 37 °C, overnight, unless stated otherwise. To investigate the influence of different carbon sources on auto-aggregation, a minimal medium was designed based on the MRS composition [73] by omitting beef extract, peptone, glucose, Tween 80 and reducing yeast extract from 0.4 to 0.2% (w/v). This minimal medium did not support *L. plantarum* growth and metabolic activity. Glucose, sucrose, maltose and fructose were added as individual carbon source at 1% (w/v). In addition, the previously designed Effluent-MacFarlane-Sugar (EMS) medium [40], mimicking the abiotic environment of in vitro colonic microbiota, was used to test the effect on auto-aggregation in absence of microbiota. EMS medium consisted of sterile filtered effluent from the in vitro colonic microbiota, MacFarlane medium [74], which mimics the chyme entering the colon (ratio 9:1), and glucose (0.75%, w/v).

### Establishment of a high-throughput screening method for auto-aggregation

A high-throughput screening method to detect auto-aggregating *L. plantarum* strains from colonic in vitro human gut microbiota was developed by modifying an existing agglutination assay [41]. MRS broth in 96-well tissue culture test plates (Bioswisstec AG, Schaffhausen, Switzerland) was inoculated with *L. plantarum* strains and grown overnight. Cultures were analyzed microscopically in biological triplicates (Leica DM1000, Leica Microsystems, Wetzlar, Germany) for aggregate formation.

### Characterization of *L. plantarum* auto-aggregation in the in vitro colonic microbiota

The design and implementation of the continuous intestinal fermentation model PolyFermS was presented in detail previously [40]. In short, the inoculum reactors (IR) of four models were inoculated (30%, v/v) with distinct fecal microbiota from healthy adults immobilized in polysaccharide gel beads. A detailed description of the PolyFermS set-up and conditions, microbiota compositions and fermentation profiles are reported in Isenring et al. [40]. The four IRs used for auto-aggregation experiments were operated for time periods ranging from 10 to 72 days. Second-stage treatment reactors (TRs) were continuously inoculated by IR effluent (5%) and fed with fresh MacFarlane medium (95%), formulated to mimic the chime entering the colon [74]. Metabolite concentrations of the continuous fermentation were determined by high-performance liquid chromatography as described previously [40]. To investigate the influence of colonic microbiota on auto-aggregation, *L. plantarum* NZ3400 was supplemented to TRs connected to the IR and operated with identical conditions. Derivatives were isolated from the effluent after 10 days from three TRs containing microbiota of donor 1, five TRs of donor 2, five TRs of donor 3 and six of donor 4.

*L. plantarum* strains were recovered from the biofilm in three reactors collected at the end of the fermentation containing microbiota of donor 2 as described previously [40] and compared to *L. plantarum* isolated from reactor effluent harvested on the same day. Nutritive stress was simulated by starvation of the modelled gut microbiota by medium inflow interruption. *L. plantarum* strains were recovered before and 24 h after starvation.

### Biofilm assay

Biofilm formation capability of the non-aggregating strain NZ3400 and its aggregating derivative strain PA4_02 was assessed in MRS, minimal medium containing glucose, sucrose, fructose or maltose as sole carbon source and EMS medium as described previously with minor modifications [75]. Wells of 96-well tissue culture test plates (Bioswisstec AG, Schaffhausen, Switzerland) containing 200 μl of corresponding growth medium were inoculated with 10^6^ CFU/ml *L. plantarum*. Wells only containing the medium served as control. The plates were incubated at 37 °C for 24 h. The liquid was removed, and the wells were washed three times with phosphate-buffered saline (PBS), pH 6.2. After drying, the wells were supplemented with crystal violet (0.1%, w/v) and incubated for 30 min. The stained biofilm was washed three times with PBS, the dye was resolved in ethanol (99%) and absorbance was measured at 595 nm (PowerWaveTMXS; Bio-Tek Instrument Inc., Winooski, VT, USA).

### Stability of auto-aggregation phenotype

The stability of auto-aggregation in 15 *L. plantarum* isolates recovered from all four microbiota was tested in three consecutive overnight cultures in MRS broth inoculated at 1% (v/v), corresponding to approximately 25 generations. Auto-aggregation was assessed visually and microscopically.

### RNA isolation and sequencing

RNA sequencing was performed on the wild type strain *L. plantarum* NZ3400, the auto-aggregating strains IA01_t0_ and PA4_02 and the non-aggregating IA01_t4_. Pre-cultures were done in MRS broth at 30 °C overnight. Experiments were performed in triplicates for NZ3400, IA01_t0_ and IA01_t4_ and in duplicates for PA4_02. Cultures were inoculated from the pre-culture and grown until OD_600nm_ = 2.6–2.7, with final measurement of the pH, glucose utilization and lactate production. Total RNA was extracted based on chloroform/phenol extraction followed by purification using the High Pure RNA isolation kit (Roche Diagnostics, Rotkreuz, Switzerland) as described previously (58). RNA quantity, purity and integrity were verified using an Agilent 2200 TapeStation (Agilent Technologies, Santa Clara, CA, USA). Samples with an RNA integrity number (RIN) > 9 and a 16S/23S-rRNA ratio > 1.5 were selected for rRNA depletion. Thereafter, EDTA was added to 1 mM and depletion was performed using the MICROBExpress™ Bacterial mRNA Enrichment Kit (Life Technologies Europe BV, Zug, Switzerland) following the manufacturer’s instructions. Concentrations of depleted samples were determined in a TapeStation and normalized to 100 ng/μl using in Tris-HCl (10 mM, pH = 8.5).

Sequencing of 100 bp single reads was done on Illumina Novaseq 6000 (Illumina Inc., California, USA) at the Functional Genomics Center Zurich (FGCZ). The library was prepared according to the Illumina Truseq Total RNA protocol.

### Analyses of DNA inversion upstream of LP_RS05225

The pipeline for the analysis of short-read re-sequencing breseq [76] was used to identify possible new junctions in the upstream region of *msa* (LP_RS05225). A 9439-bp fragment of the WCFS1 genome 7000 bp upstream and 2000 bp downstream of the *marR* gene (LP_RS05230) was used as reference and the RNAseq reads as re-sequencing reads. Standards settings were applied, and each set of reads was analyzed separately.

To quantify the amount of standard and reversed promoter regions of *msa* in the strains, a fragment containing the 234-bp upstream regions of the *msa* start with both promotor orientations was constructed and stored as two sequences in a single fasta file. RNAseq reads were plotted to the sequences using Bowtie2 [77] with standard settings. The Bowtie2 output was further processed to a reads-per-gene spreadsheet as described above. The ratio between both promoter regions was calculated and averaged. A two-tailed t-test was used to calculate the *p* value.

To identify a possible subpopulation harboring the *msa-MarR*-inversion, primers were designed (Additional file 8: Figure S4) to amplify the native sequence (Native_fw: 5′-GGGAGTAAAGCGTGCAATGT-3′; Native_rev: 5′-GCATTACCTATTTGATAACGCAGA-3′) and the inverted sequence (Inversion_fw: 5′-TCATGCGAAAGGATAGGTGTAA-3′; Inversion_rev:5′- TTGAGATGCTGAATCGTTCG-3′) in the promoter region. DNA of the non-aggregating wild type NZ3400 and IA10, and the aggregating PA4_02 and IA01 was extracted as described previously using lysozyme-cell-lysis and purification using the Wizard Genomic DNA purification kit (Promega, Dübendorf, Switzerland) [40]. PCR reactions were performed in a volume of 25 μl containing 20 ng DNA, 12.5 μl 2 x PCR Master Mix (Fermentas, Le Mont-sur-Lausanne, Switzerland), 1 μM of each primer (Microsynth, Balgach, Switzerland) and sterile, DNase-free water (Fermentas). PCR was performed with an initial denaturation (95 °C, 2 min), followed by 30 or 40 cycles of denaturation (95 °C, 30 s), annealing (51 °C, 30 s) and replication (72 °C, 25 s) and subsequent final replication (72 °C, 7 min) in a Biometra® T3000 Thermocycler (Labgene, Châtel-Saint-Denis, Switzerland). Amplified products and DNA marker (100 bp, BioConcept, Allschwil, Switzerland) were analyzed via agarose (2%, w/v) gel electrophoresis and visualized with gel red.

### Data analysis

Graphs were created using GraphPad Prism® version 8 (GraphPad Software Inc., San Diego, CA, USA). Analysis of RNAseq reads was performed as described previously [78]. Shortly, reads were mapped on the chromosome and plasmid of *L. plantarum* WCFS1 (accession numbers NC_006375.1, NC_006376.1, NC_006377.1, and NC_004567.2) using Bowtie2. Data filtering, normalization and analysis was done in R (version 3.6.2) using the packages DESeq and EdgeR. Only genes with a false discovery rate (FDR) < 0.05 and a differential expression of minimum 2 fold (│ratio(log_2_) > 1│) were considered as significant differently expressed.

## Supplementary Information

**Additional file 1: Figure S1.** Biofilm formation ability of the non-aggregating wild type strain *L. plantarum* NZ3400 (blue) and the isogenic aggregating strain PA4_02 (red). Biofilm formation was assessed in MRS and EMS medium and minimal medium containing sucrose (mm-sucrose), fructose (mm-fructose), glucose (mm-glucose) or maltose (mm-maltose) as carbon source. Data represent mean value ± standard deviation of biological triplicates.

**Additional file 2: Figure S2.** Fermentation metabolite profile before (blue) and after (red) microbiota starvation. Bars represent mean ± standard deviation of metabolite abundance relative to the total produced metabolites of six parallel operated TRs the day before and 24 h after starvation of the gut microbiota. Significance was calculated by paired-sample *t* test: ** < 0.01, **** *p* < 0.0001.

**Additional file 3: Table S1.** Significantly up- and downregulated genes in the auto-aggregating *L. plantarum* IA01 compared to NZ3400.

**Additional file 4: Table S2.** Significantly up- and downregulated genes in the auto-aggregating *L. plantarum* IA01t_0_ compared to the non-aggregating IA01t_4_.

**Additional file 5: Table S3.** Significantly up- and downregulated genes in the auto-aggregating *L. plantarum* PA4_02 compared to NZ3400.

**Additional file 6: Table S4.** Significantly up- and downregulated genes in all auto-aggregating *L. plantarum*.

**Additional file 7: Figure S3.** Visualization of DNA fragments (agarose gel, 2%) obtained by PCR amplification using 30 (A) and 40 (B) cycles of denaturation, annealing and replication. NZ3400: *L. plantarum* wild type strain (non-aggregating); IA10: *L. plantarum* recovered from the gut microbiota (non-aggregating); PA4_02 and IA01: *L. plantarum* recovered from the gut microbiota (auto-aggregating).

**Additional file 8: Figure S4.** Scheme of the PCR-set-up to detect the native and inversed *msa-marR* promoter region. A) Native orientation: DNA will be amplified using the primer combination Native_fw/Native_rev, resulting in a 225 bp amplicon. B) Inverted DNA will be amplified using the primer combination Inverse_fw/Inverse_rev, resulting in a 313 bp amplicon. P: promoter.

## Data Availability

Gene expression data are directly accessible through GEO (series accession number GSE172351, https://www.ncbi.nlm.nih.gov/geo/query/acc.cgi?acc=GSE172351).
