# Supplementary figures and images for: Bistable auto-aggregation phenotype in Lactiplantibacillus plantarum emerges after cultivation in in vitro colonic microbiota
Source: BMC Microbiol. 2021 Oct 5;21:268. doi: 10.1186/s12866-021-02331-x (PMC8493755; doi:10.1186/s12866-021-02331-x)

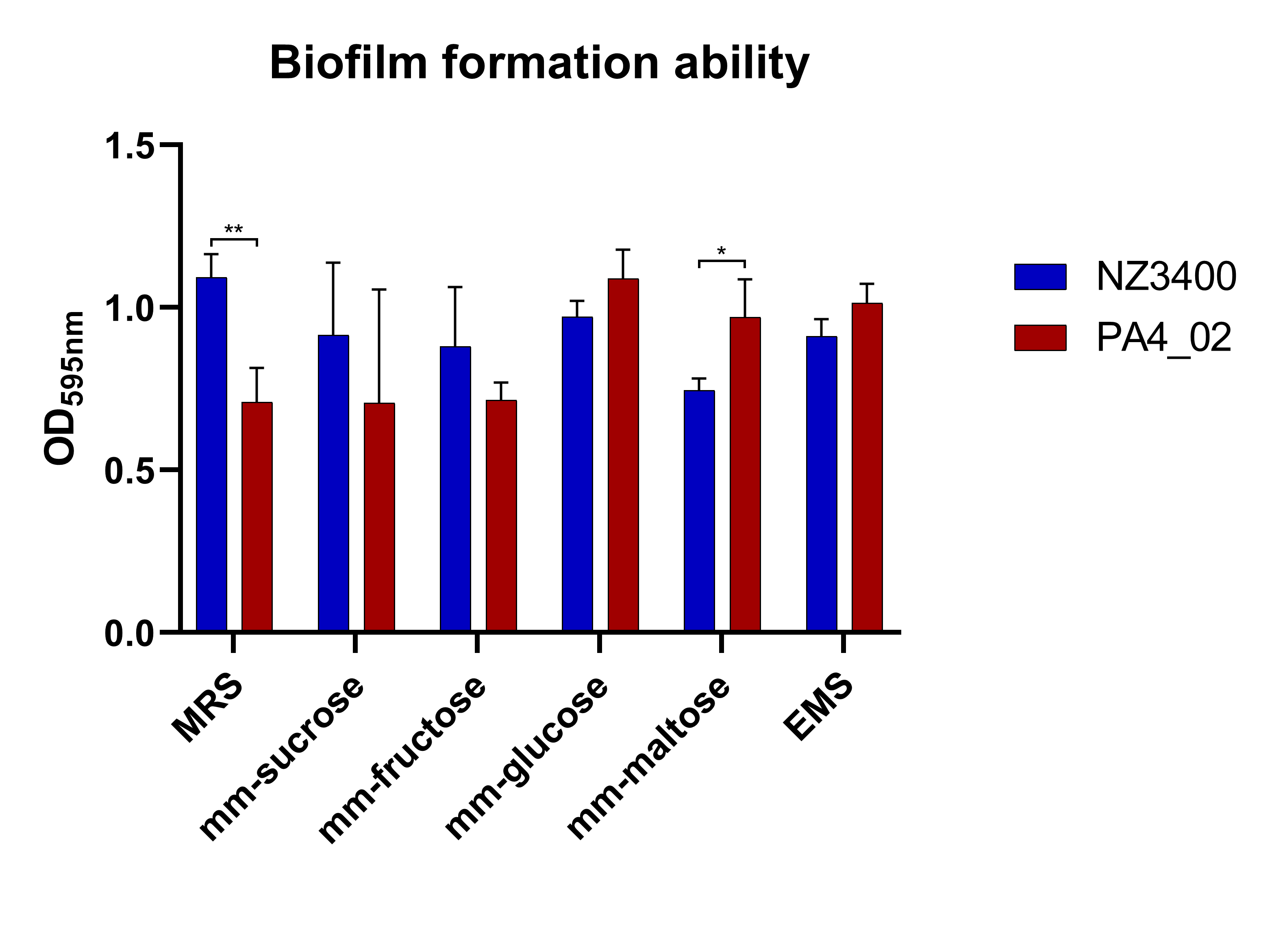

Supplement: Supplementary file 1 — Additional file 1: Figure S1. Biofilm formation ability of the non-aggregating wild type strain L. plantarum NZ3400 (blue) and the isogenic aggregating strain PA4_02 (red). Biofilm formation was assessed in MRS and EMS medium and minimal medium containing sucrose (mm-sucrose), fructose (mm-fructose), glucose (mm-glucose) or maltose (mm-maltose) as carbon source. Data represent mean value ± standard deviation of biological triplicates. [file 12866_2021_2331_MOESM1_ESM.tif]

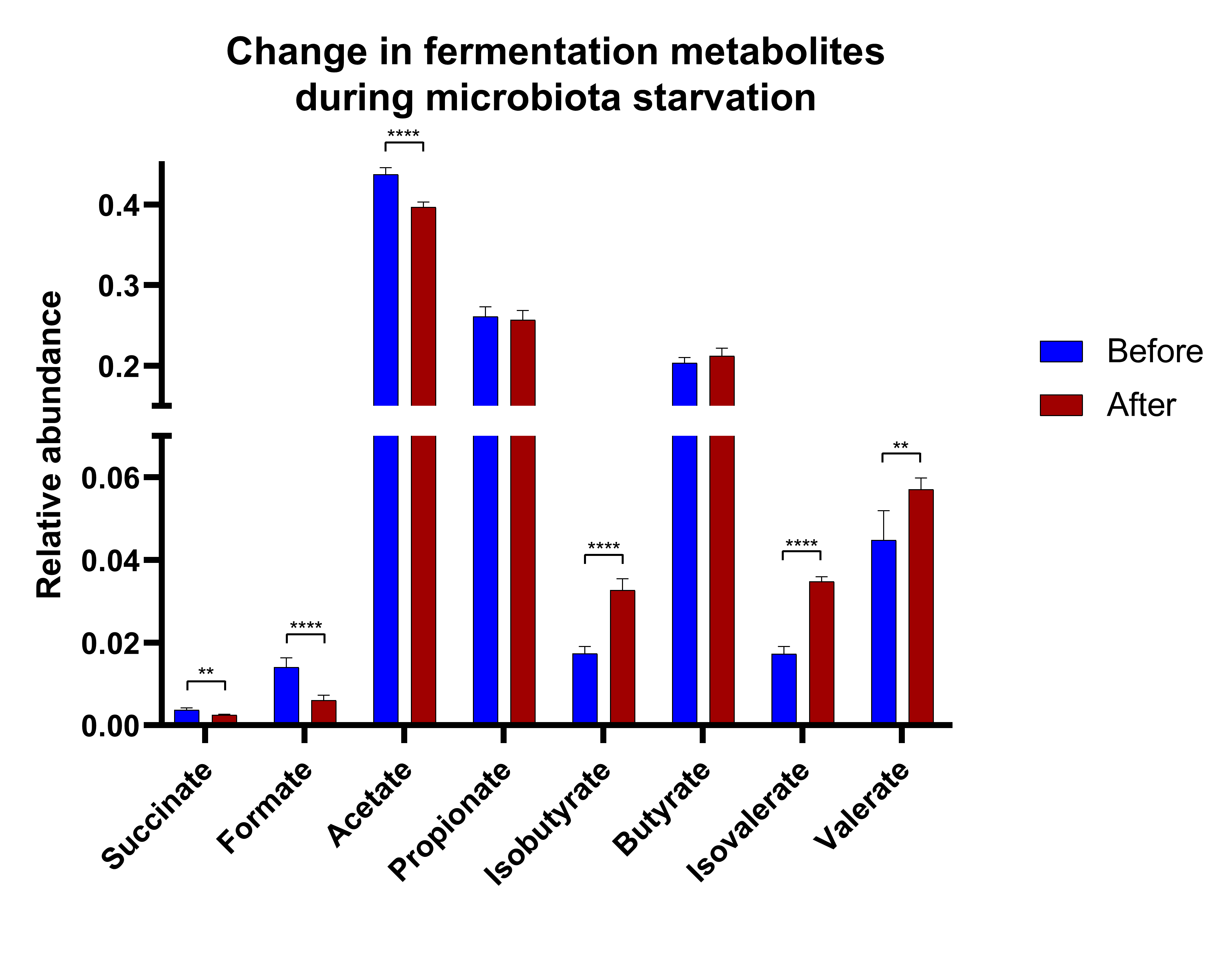

Supplement: Supplementary file 2 — Additional file 2: Figure S2. Fermentation metabolite profile before (blue) and after (red) microbiota starvation. Bars represent mean ± standard deviation of metabolite abundance relative to the total produced metabolites of six parallel operated TRs the day before and 24 h after starvation of the gut microbiota. Significance was calculated by paired-sample t test: ** < 0.01, **** p < 0.0001. [file 12866_2021_2331_MOESM2_ESM.tif]

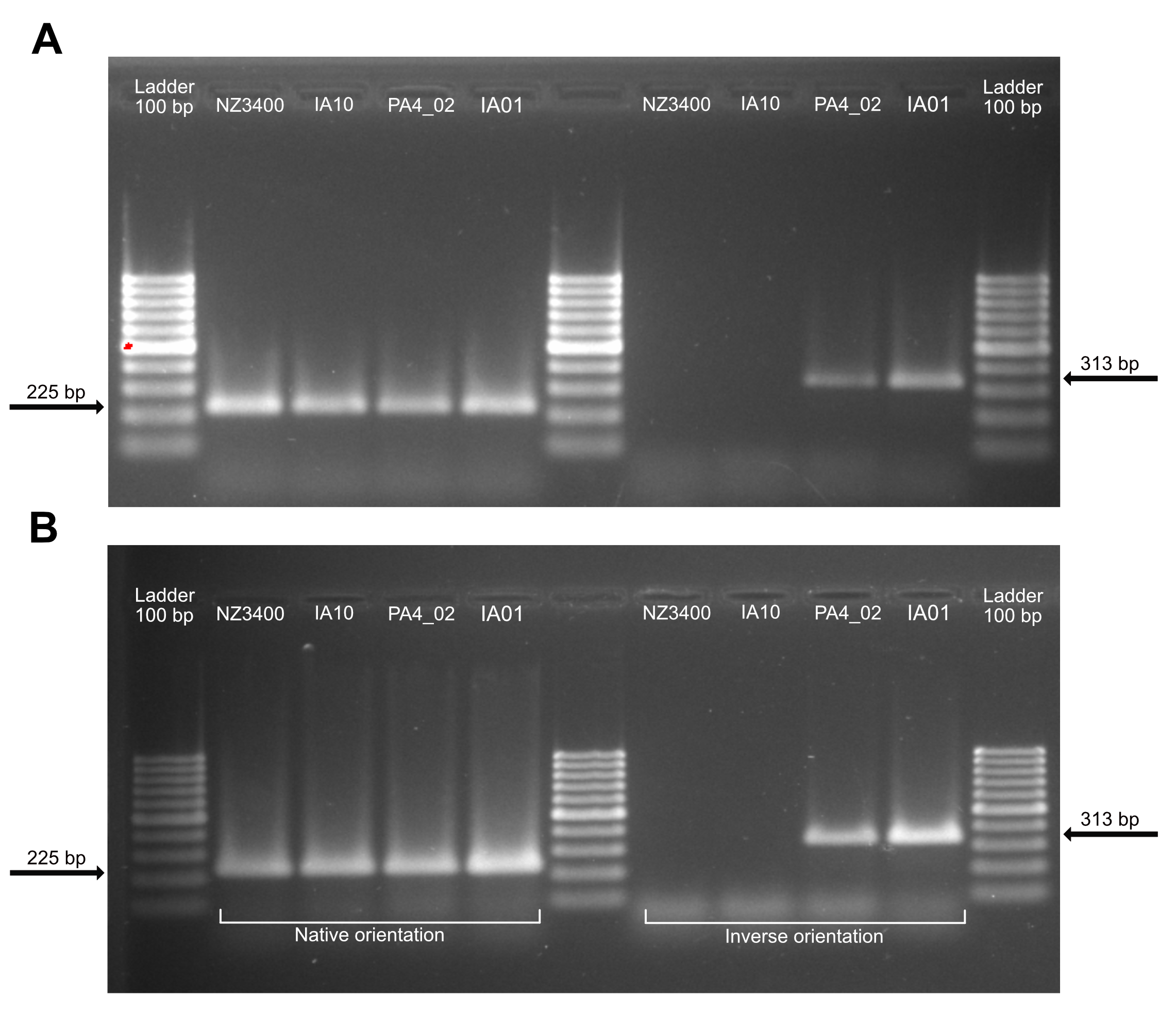

Supplement: Supplementary file 7 — Additional file 7: Figure S3. Visualization of DNA fragments (agarose gel, 2%) obtained by PCR amplification using 30 (A) and 40 (B) cycles of denaturation, annealing and replication. NZ3400: L. plantarum wild type strain (non-aggregating); IA10: L. plantarum recovered from the gut microbiota (non-aggregating); PA4_02 and IA01: L. plantarum recovered from the gut microbiota (auto-aggregating). [file 12866_2021_2331_MOESM7_ESM.tiff]

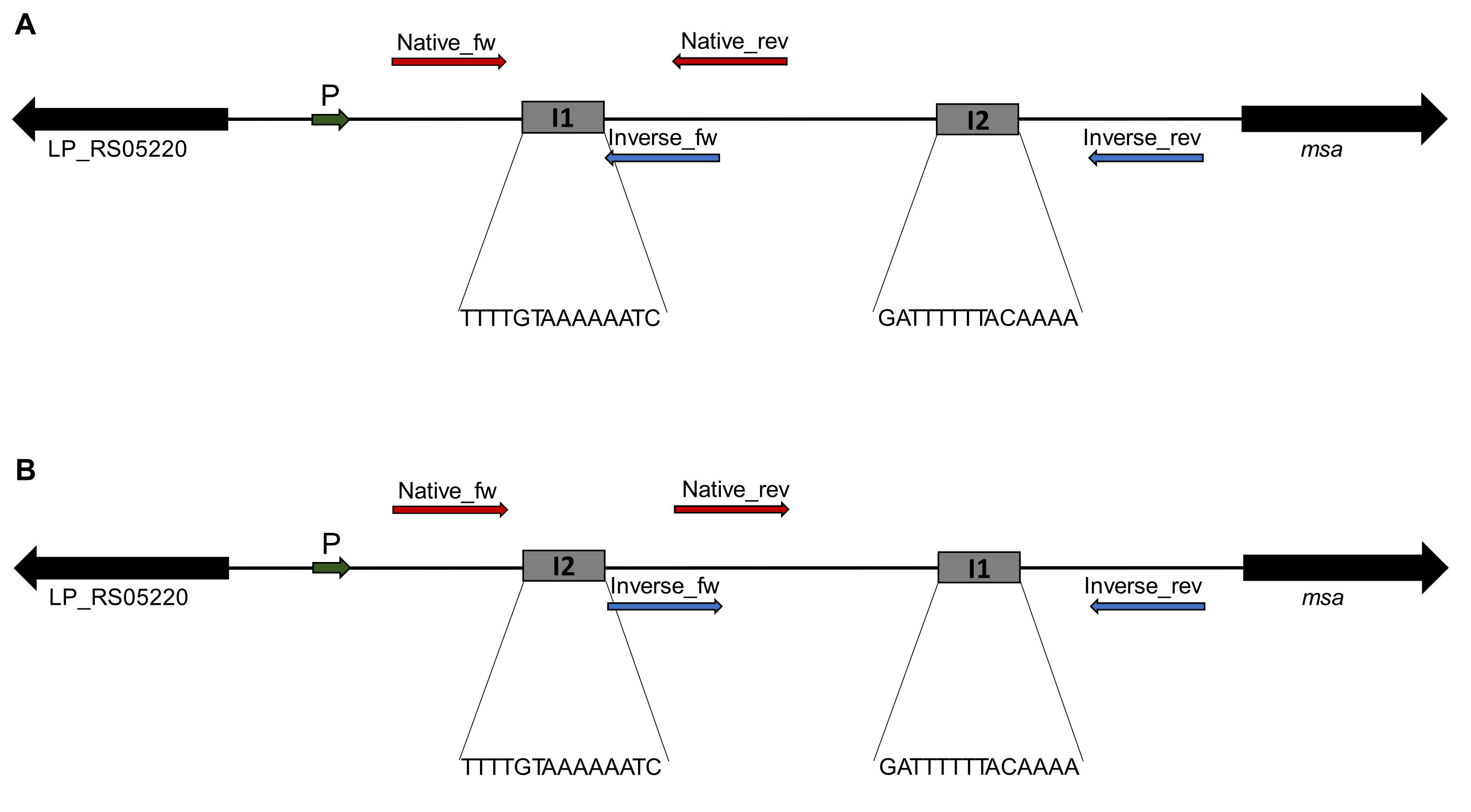

Supplement: Supplementary file 8 — Additional file 8: Figure S4. Scheme of the PCR-set-up to detect the native and inversed msa-marR promoter region. A) Native orientation: DNA will be amplified using the primer combination Native_fw/Native_rev, resulting in a 225 bp amplicon. B) Inverted DNA will be amplified using the primer combination Inverse_fw/Inverse_rev, resulting in a 313 bp amplicon. P: promoter. [file 12866_2021_2331_MOESM8_ESM.tiff]
